# Supplementary material for: Mechanically driven Li dendrite penetration in garnet solid electrolyte
Source: Nature. 2026 Apr 22;652(8111):912–8. doi: 10.1038/s41586-026-10415-9 (PMC13102695; doi:10.1038/s41586-026-10415-9)
Supplement: Supplementary file 1 — This file contains Supplementary Table 1, Supplementary Figs. 1–34 and Supplementary References. [file 41586_2026_10415_MOESM1_ESM.pdf]

nature portfolio

https://doi.org/10.1038/s41586-026-10415-9

Supplementary information

Mechanically driven Li dendrite penetration in garnet solid electrolyte

In the format provided by the authors and unedited

Supplementary Information for

Mechanically Driven Li Dendrite Penetration in Garnet Solid Electrolyte

Yuwei Zhang*, Soroush Motahari, Eric V. Wood, Stefan Zaefferer, Peter Schweizer, Zhiyuan Zhang, Yuqi Liu, Baptiste Gault, Franz Roters, Dierk Raabe, Christina Scheu, Yug Joshi, Siyuan Zhang*, Chuanlai Liu*, Gerhard Dehm*

*Corresponding authors. Email: yuwei.zhang@mpi-susmat.de, siyuan.zhang@mpi-susmat.de, c.liu@mpi-susmat.de, dehm@mpi-susmat.de

The PDF file includes:

Table S1
Figs. S1 to S34

Other Supplementary Materials for this manuscript include the following:

Movie S1

Table S1. Parameters used in the simulations.

Mechanics	Isotropic LLZTO elastic parameters	C11	183.5×10^9 (Pa)	Ref [1]
		C12	64.5×10^9 (Pa)	
		C44	59.5×10^9 (Pa)	
	Anisotropic LLZTO elastic parameters	C11	169.8×10^9 (Pa)	Ref [2]
		C12	63.9×10^9 (Pa)	
		C44	69.8×10^9 (Pa)	
	Isotropic lithium elastic parameters	C11	13.34×10^9 (Pa)	Ref [3]
		C12	11.19×10^9 (Pa)	
		C44	8.83×10^9 (Pa)	
	Lithium plasticity parameters	$\dot{\gamma}_0$	10^{-3} (s^{-1})	Bayesian optimization based on Ref [4]
		n	6.6	
		ξ_0	2.6×10^3 (Pa)	
		ξ_∞	4.5×10^3 (Pa)	
		a	2.0	
		h_0	2.1×10^6 (Pa)	
		M	3.0	
		c_1	8.05	
		c_2	2.92	
		c_3	5.97	
Phase-field	Damage	$\mathcal{G}_c$	6.22 (Jm^{-2})	Ref [5, 6]
		l_0	4×10^{-6} (m)	
		M	2.57×10^{-4} ($m^3 J^{-1} s^{-1}$)	
	Reaction parameters	$\dot{\eta}$	0.06 (s^{-1})	
		$\Omega\eta_{max}$	1	Ref [1]
		Ω	13×10^{-6} ($m^3 mol^{-1}$)	Ref [1]
Model set-up		Grid size	1×10^{-6} (m)	N/A
		Model discretization	128×256 2D<br>$128 \times 256 \times 10$ 3D	
		Time increment	5×10^{-4} (s)	

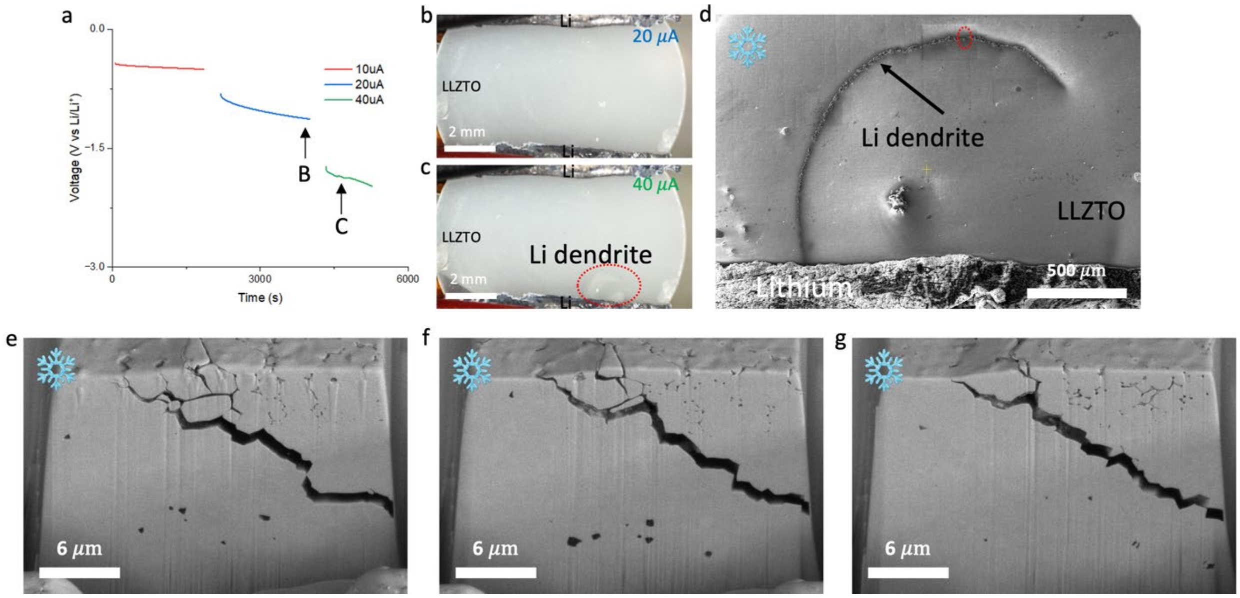

Figure S1. Morphology of lithium dendrite growth in 1 mm-thick LLZTO solid electrolyte. (a) Voltage response at different currents. (b) & (c) Top-view optical microscope images of lithium dendrite growth at different stages, as indicated in (a). (d) Cryogenic SEM image of the lithium dendrite as circled in in (c). (e–g) Cross-sectional images of lithium dendrite growth in the solid electrolyte at the location highlighted by the red circle in (d). Data source: in-plane cell.

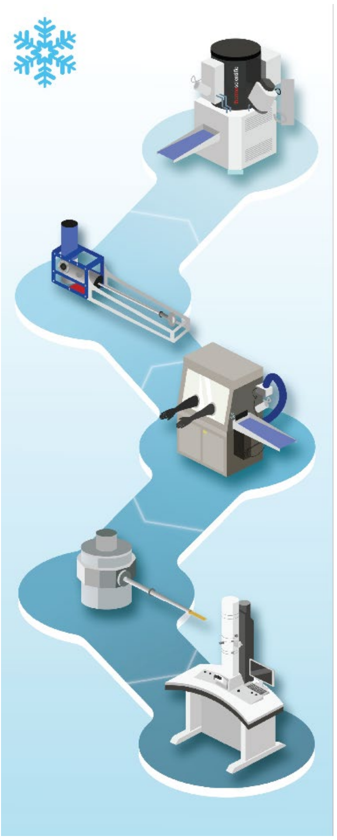

Figure S2. Schematic of the cryogenic sample preparation and characterization workflow, from the glovebox to cryogenic FIB, TKD, EBSD in a scanning electron microscope (SEM) and electron energy loss spectroscopy (EELS), diffraction imaging in scanning transmission electron microscopy (STEM), using an inert/vacuum transfer holder.

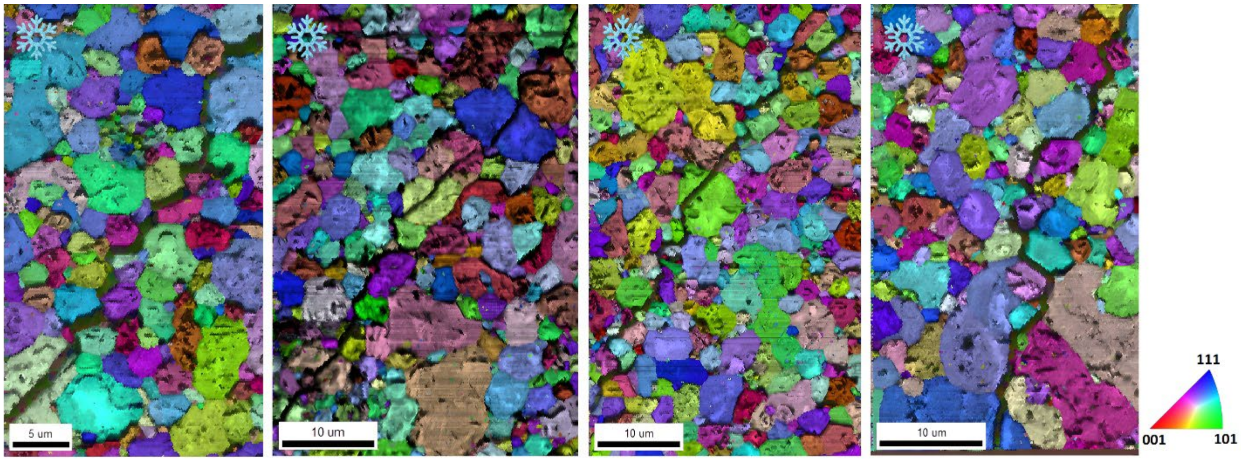

Figure S3. EBSD pattern of lithium plating-induced fracture in the LLZTO solid electrolyte. Data source: in-plane cell.

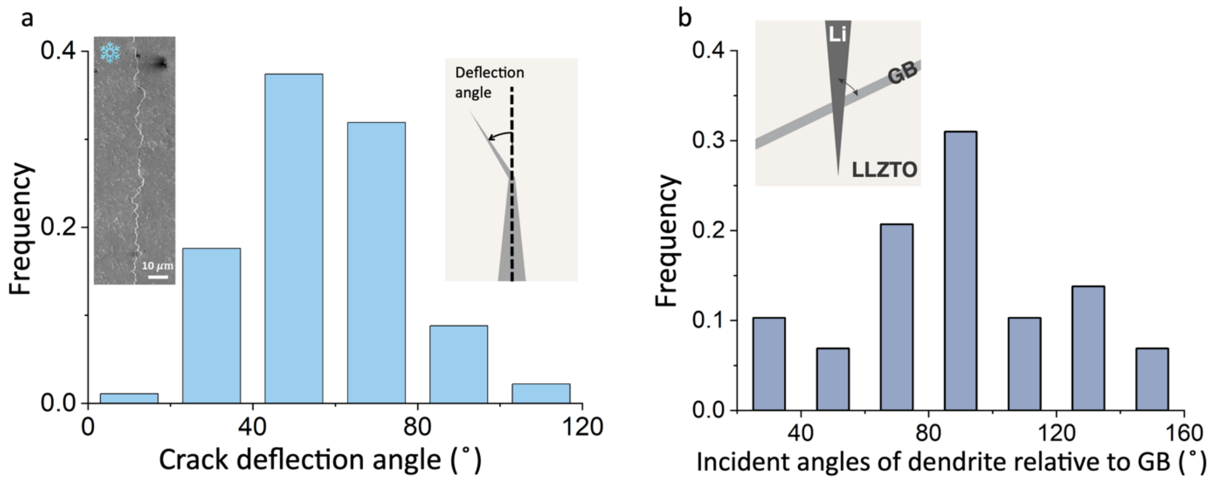

Figure S4. (a) Statistical frequency distribution of crack deflection angles based on SEM imaging data. (b) Statistical frequency distribution of the incident angle between the grain boundary and the lithium dendrite trajectory at transgranular fracture sites. Data source: in-plane cell.

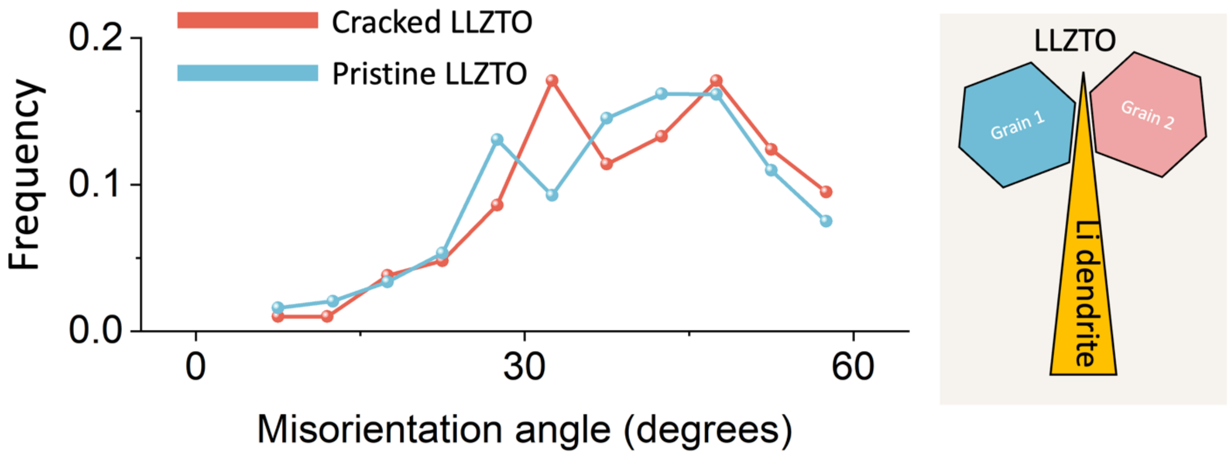

Figure S5. Crystal lattice misorientation angle distribution along intergranular fracture paths in LLZTO, defined as the misorientation between adjacent Grain 1 and Grain 2 as shown in the schematic. This distribution is compared with the grain boundary misorientation distribution in pristine LLZTO. Approximately 100 grain boundaries were analyzed. Data source: in-plane cell.

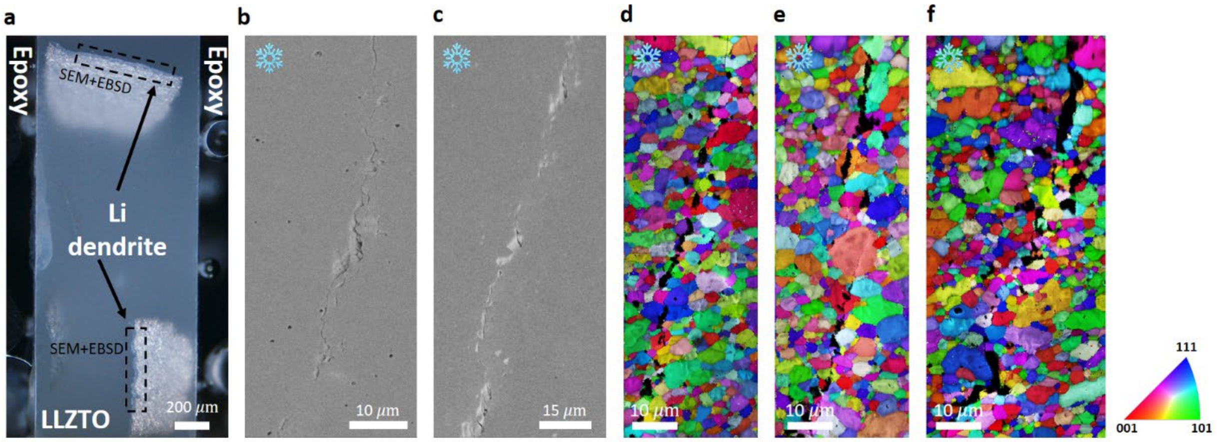

Figure S6. (a) Optical microscopy image highlighting the region selected for cryogenic SEM and EBSD characterization. (b, c) SEM images of a short-circuited LLZTO solid electrolyte prepared under a symmetric cell configuration. (d-f) EBSD maps showing Li plating-induced fracture in the LLZTO solid electrolyte under the same configuration. Data source: symmetrical cell.

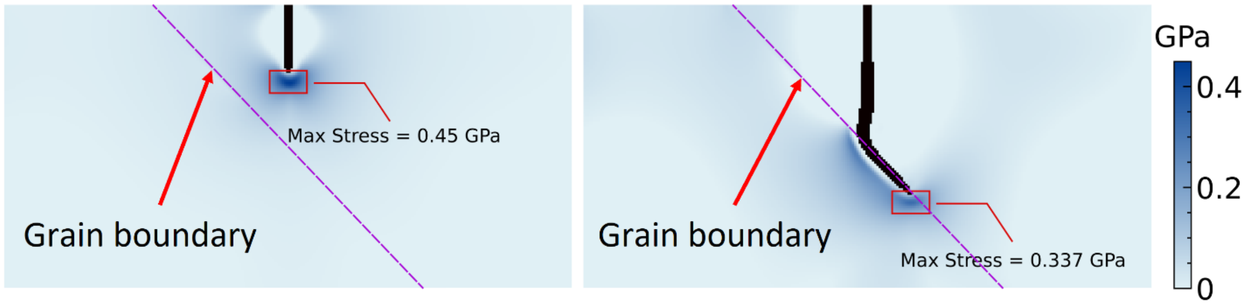

Figure S7. Evolution of the maximum tensile stress in the solid electrolyte after the lithium dendrite encounters a grain boundary with a deflection angle of 45 degrees.

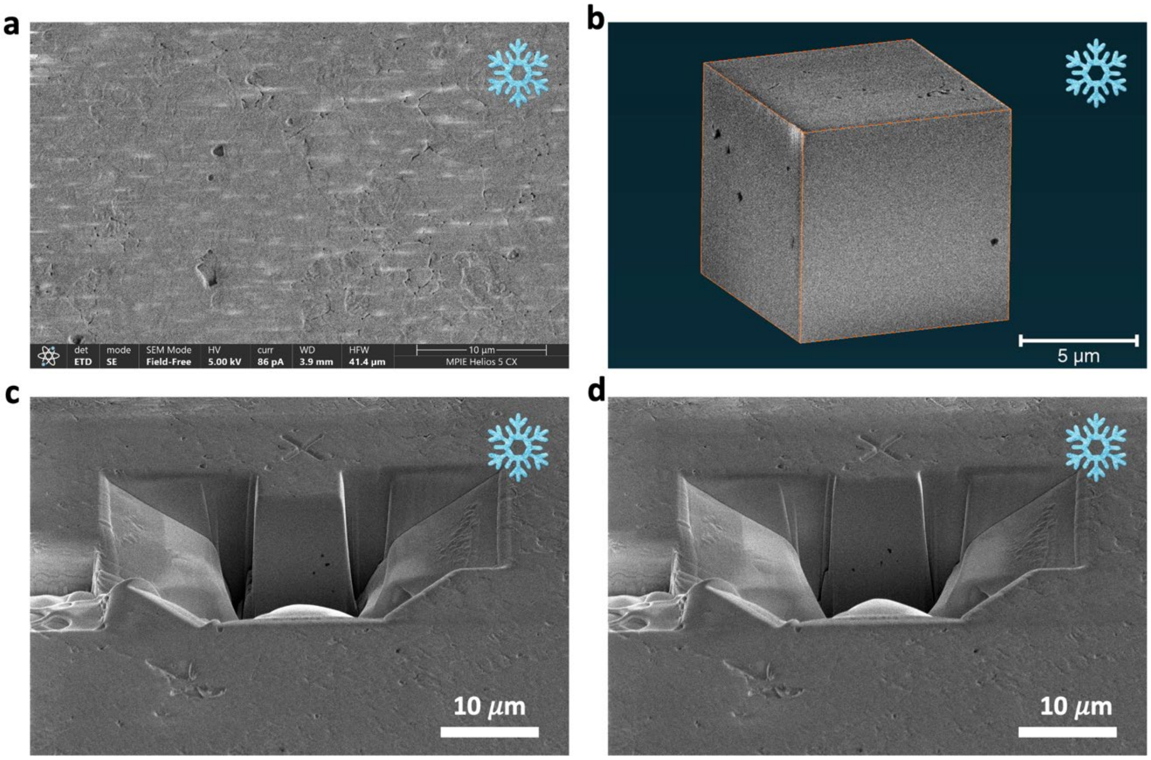

Figure S8. Morphology of mechanically polished LLZTO after 30 s acid treatment. (a) Top view of the LLZTO solid electrolyte. (b) 3D reconstruction of the LLZTO solid electrolyte; (c, d) Representative slices from the 3D reconstructed volume.

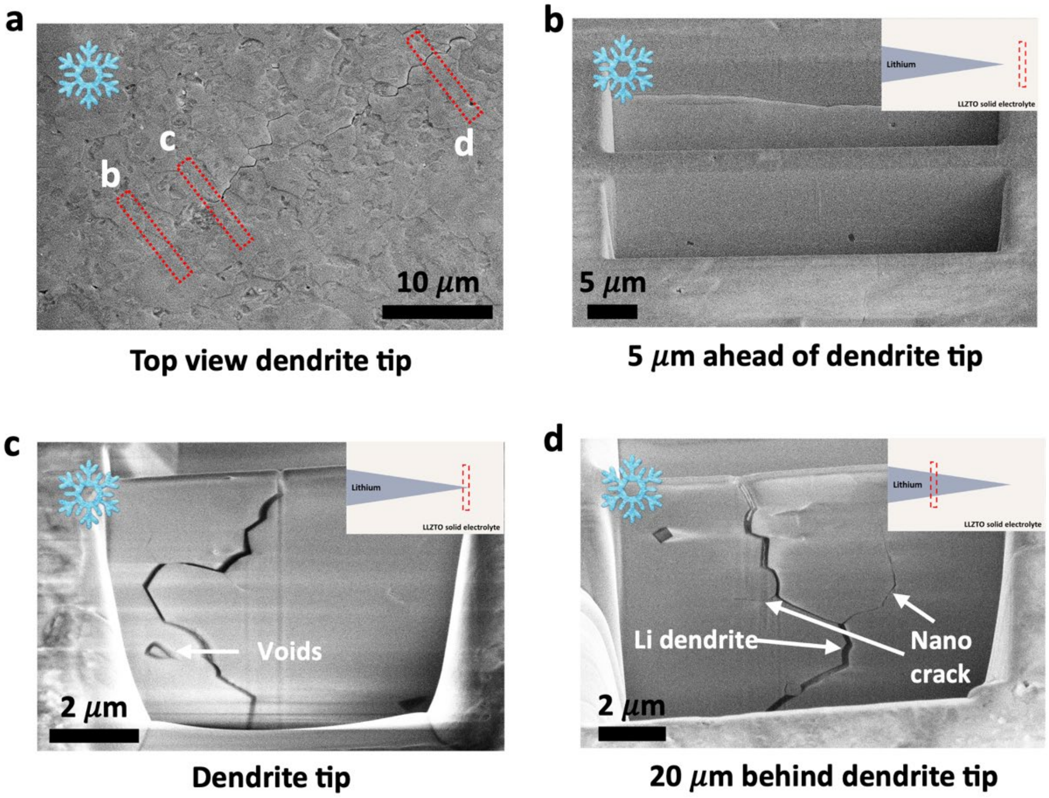

Figure S9. Morphology of the lithium dendrite tip. (a) Top-view image of the lithium dendrite tip within the LLZTO solid electrolyte. (b-d) Cross-sectional fractography of LLZTO induced by lithium plating, captured at different positions as labelled in (a). (b) 5 μm ahead of the dendrite tip, (c) exactly at the dendrite tip, and (d) 20 μm behind the dendrite tip. Data source: in-plane cell.

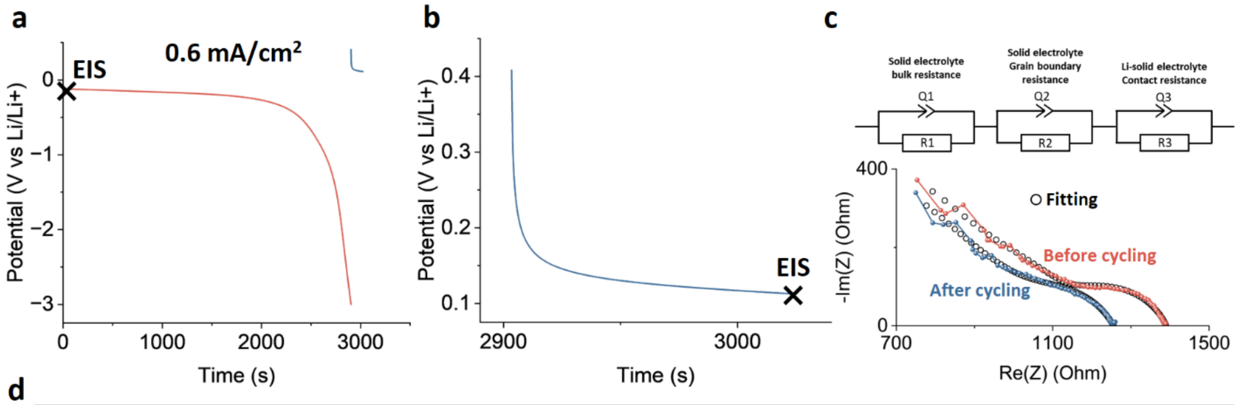

d

Before cycle	Q_bulk	a_bulk	R_bulk		Q_gb	a_gb	R_gb		Q_interface	a_interface	R_interface	
Unit	F	/	ohm	ohm*cm^2	F	/	ohm	ohm*cm^2	F	/	ohm	ohm*cm^2
Value	9.73E-12	1	823.8	174.9751	7.00E-09	0.80	302.2	64.2	4.45E-07	0.72	265.0	56.3

After cycle	Q_bulk	a_bulk	R_bulk		Q_gb	a_gb	R_gb		Q_interface	a_interface	R_interface	
Unit	F	/	ohm	ohm*cm^2	F	/	ohm	ohm*cm^2	F	/	ohm	ohm*cm^2
Value	9.43E-12	1	750.0	159.3	1.20E-08	0.75	259.1	55.0	3.00E-07	0.69	246.3	52.3

Figure S10. (a) Potential response during lithium plating and stripping in the symmetric cell at 0.6 mA/cm^2. The cell was shut down after 600 s during the stripping stage. (b) Enlarged view of the stripping period. (c) Electrochemical impedance spectroscopy results before and after cycling. (d) Fitting parameters for EIS data. Data source: symmetrical cell.

Notes for Figure S10. As shown in the equivalent-circuit fitting, the interfacial response between lithium and LLZTO shows a non-ideal capacitive feature and is described as a constant phase element (CPE) with an exponent of a=0.82. For a constant phase element (a < 1), the imaginary component is intrinsically reduced relative to the real component, leading to a depressed or flattened arc rather than a perfect semicircle. The CPE behavior of the Li-LLZTO interface is due to a continuous spatially heterogeneous charge and mass transfer.[7, 8] Such compressed semicircular features have been widely reported for Li-garnet electrolyte interfaces.[9-12]

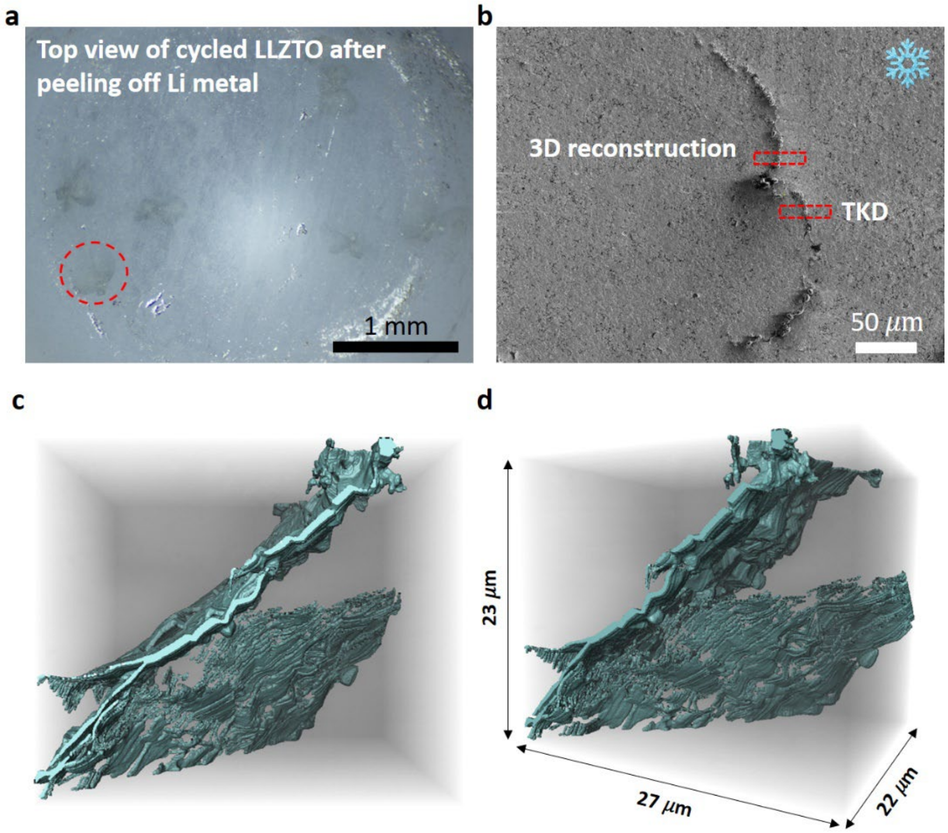

Figure S11. (a) Top-view optical microscopy image of the cycled solid electrolyte corresponding to the cycling curve in Figure S10a. The red circle marks the region selected for electron microscopy characterization. (b) Magnified view of the cracked region used for subsequent analysis. (c, d) Three-dimensional reconstruction of Li dendrites in the solid electrolyte grown in the symmetric cell configuration, displayed from two different viewing angles. Data source: symmetrical cell.

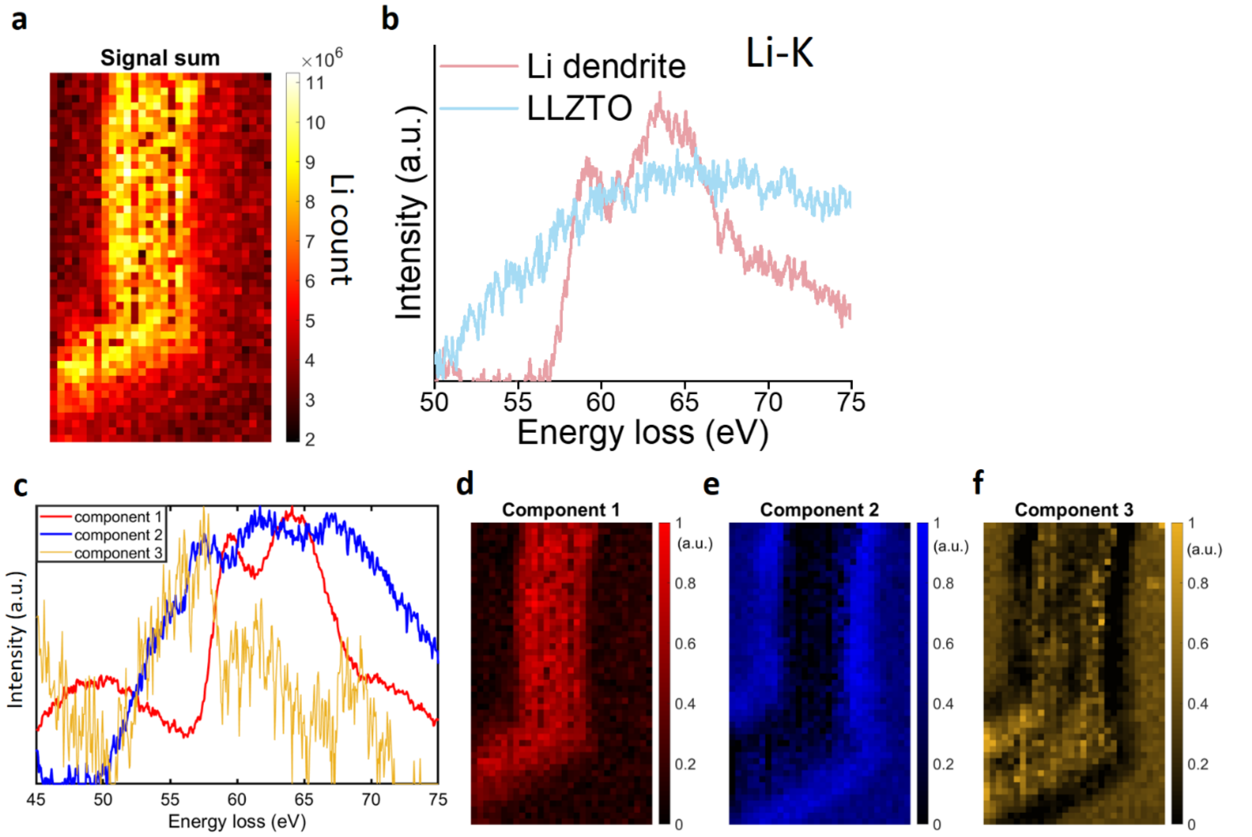

Figure S12. (a) EELS Li count map at the lithium dendrite tip (same dataset as in Fig. 2g). (b) Integrated EELS spectra from LLZTO and the lithium dendrite obtained from the raw EELS dataset. The spectrum from the lithium dendrite exhibits characteristic features of LiOH, indicating contamination introduced during sample transfer from cryo-FIB to cryo-STEM. TKD-SEM measurement results performed prior to transfer confirm that the lithium dendrite consisted of pristine metallic lithium, as supported by the high confidence index in the EBSD analysis in Fig. 3b. Accordingly, throughout the manuscript, LiOH-related EELS peaks are attributed to contamination of originally pristine lithium. (c-f) Multivariate statistical analysis applied to the dataset in Fig. S12a, showing the three leading spectral components (c) and their corresponding weighting maps (d-f). Further methodological details are provided in the Methods section (Cryogenic STEM). Data source: in-plane cell.

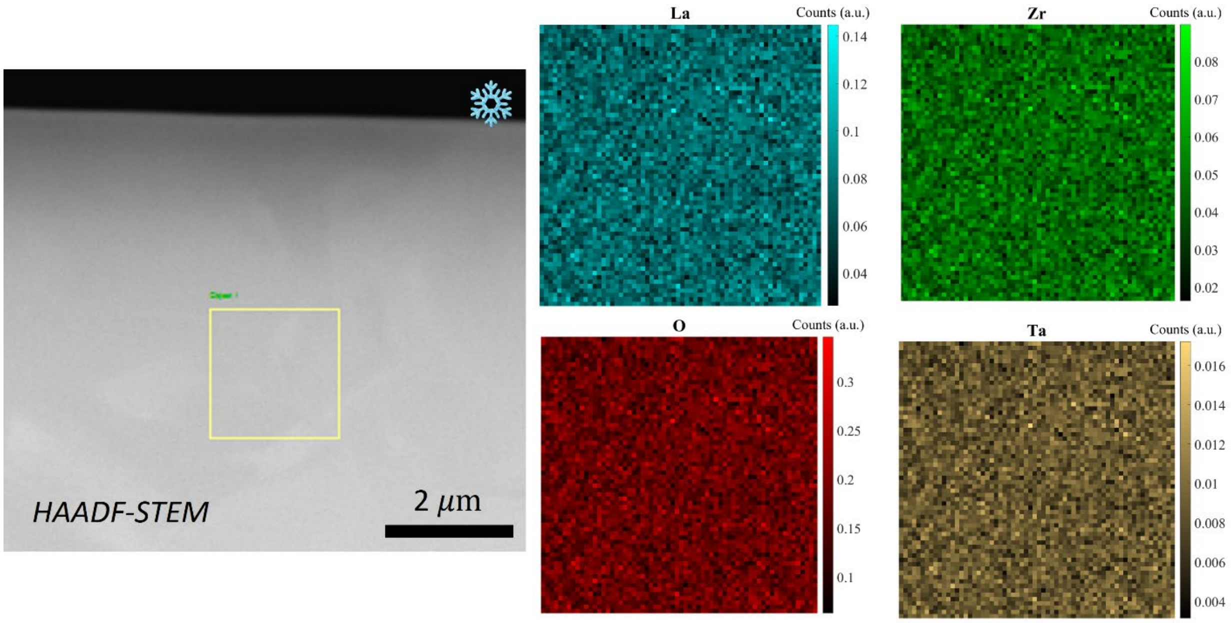

Figure S13. A STEM lamella prepared approximately 1 µm ahead of the Li dendrite tip, showing the corresponding HAADF-STEM and EDS results. The EDS scan covers an area of 2 µm × 2 µm. Data source: in-plane cell.

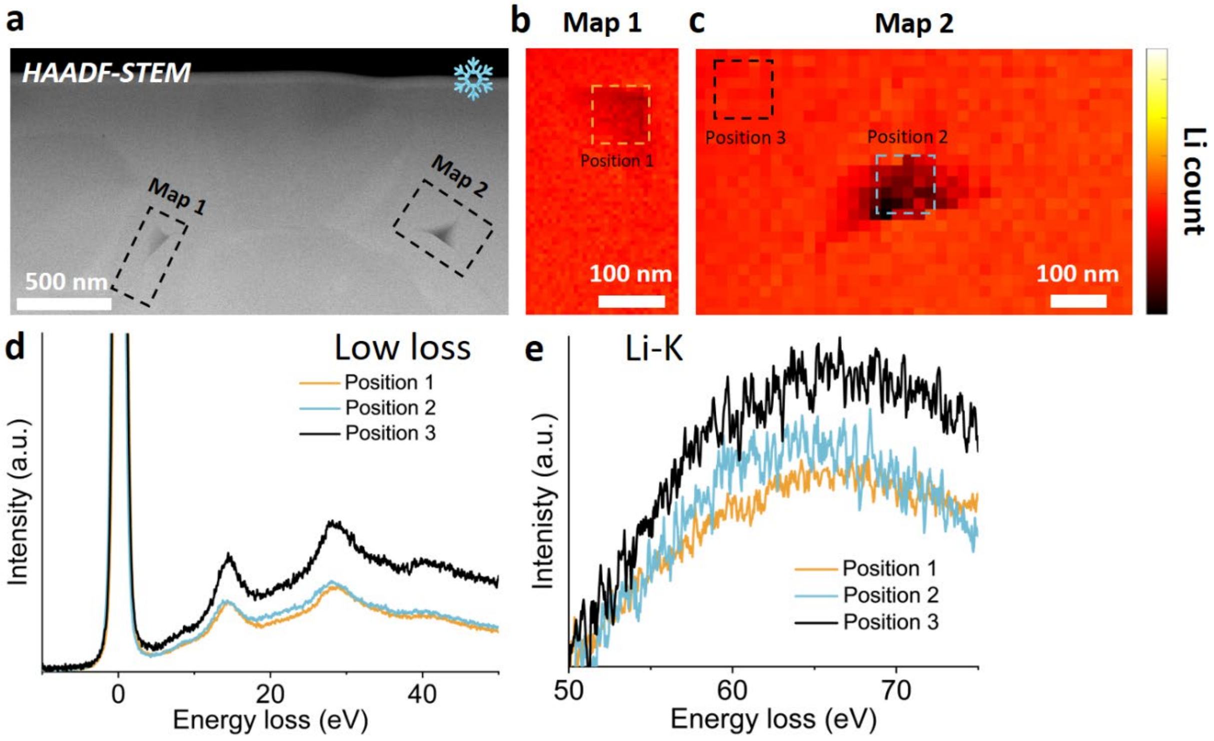

Figure S14. (a) Cross-sectional observation of a region located approximately 5 μm ahead of the dendrite tip. The size and geometry of the triple junctions exhibit features similar to those in pristine LLZTO, as shown in Figures S8. (b, c) Corresponding EELS lithium count maps of the boxed regions in (a), confirming the absence of lithium ahead of the dendrite tip. EELS spectra of (d) low loss and (e) Li-K edge. Data source: in-plane cell.

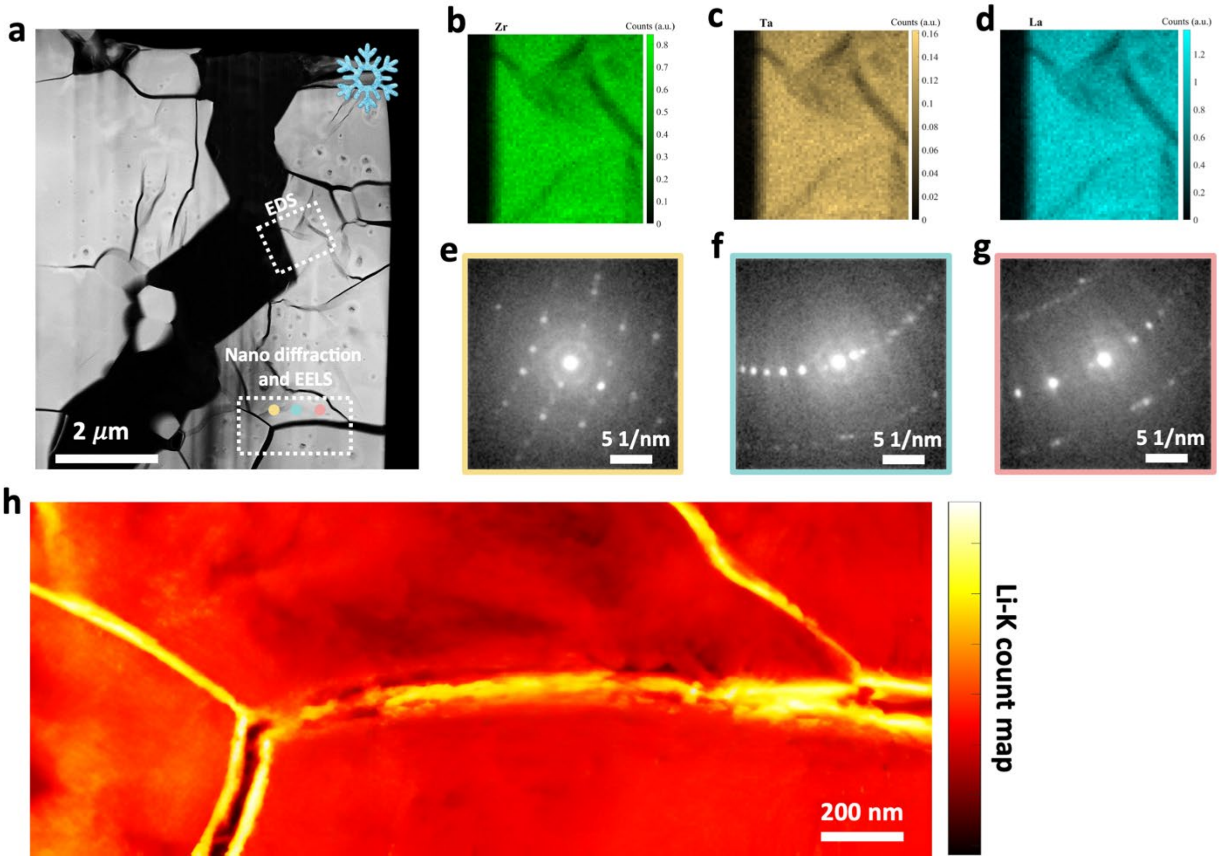

Figure S15. Chemical and microstructural characterization of a widened lithium dendrite within the solid electrolyte. (a) HAADF-STEM image of the lithium dendrite. (b-d) EDS results for the regions highlighted in (a). (e-g) Nano-diffraction patterns corresponding to the regions marked in (a), which are located approximately 300 nm above the horizontal Li dendrite. (h) EELS mapping showing lithium counts for the areas indicated in (a). Data source: in-plane cell.

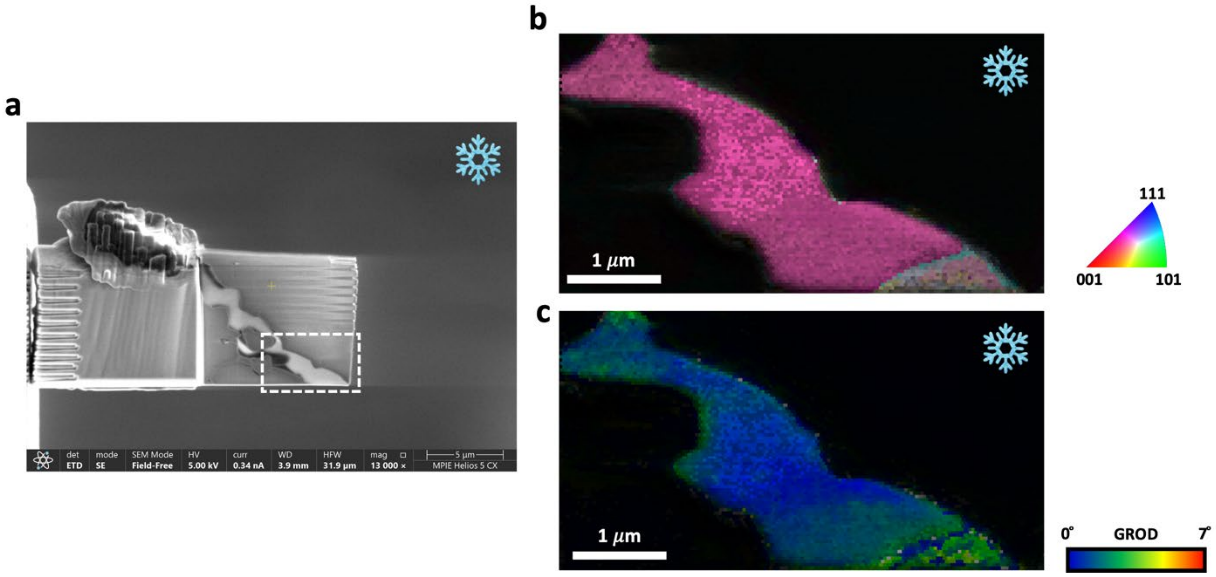

Figure S16. Lattice orientation analysis of a lithium dendrite within the LLZTO solid electrolyte prepared through the in-plane cell. (a) SEM image of a lithium dendrite. (b) Lattice orientation map of the lithium dendrite. (c) Grain reference orientation deviation angle map of the lithium dendrite. Data source: in-plane cell.

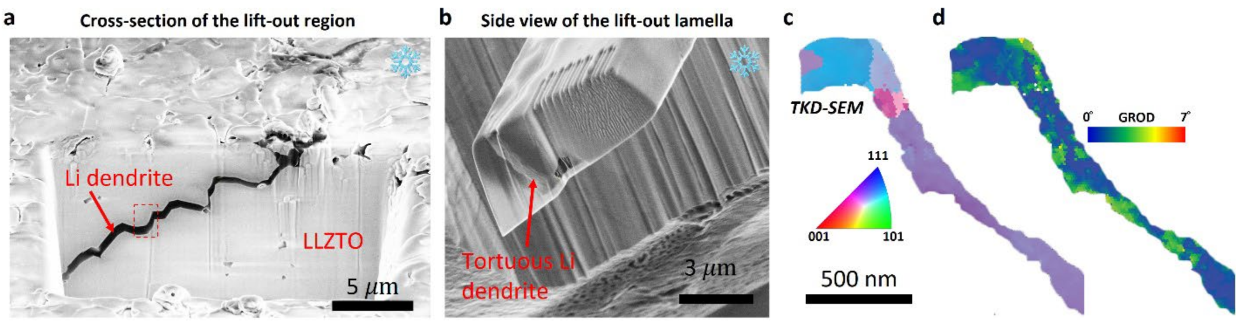

Figure S17. (a) Cross-sectional view of a lithium dendrite penetrating the solid electrolyte after electrochemical cycling in the symmetric cell configuration. The red square marks the region selected for TKD measurement. (b) Side view of the lift-out lamella prepared for TKD analysis, showing that the lithium dendrite exhibits a tortuous three-dimensional morphology. (c) TKD-SEM map showing the grain orientations of the lithium dendrite. (d) Grain reference orientation deviation map of the same region. Data source: symmetrical cell.

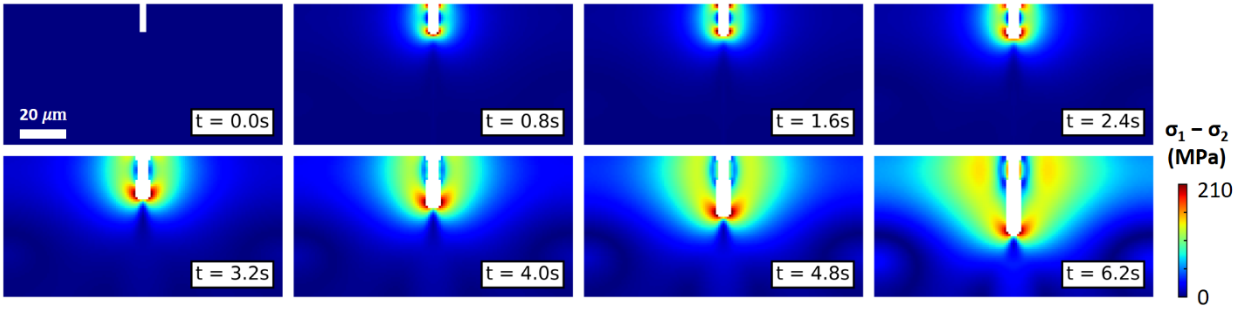

Figure. S18. Distribution of the difference between the principal stresses $\sigma_1 - \sigma_2$ during lithium dendrite propagation. The simulation results show good agreement with the experimental measurement reported in a previous literature.[13]

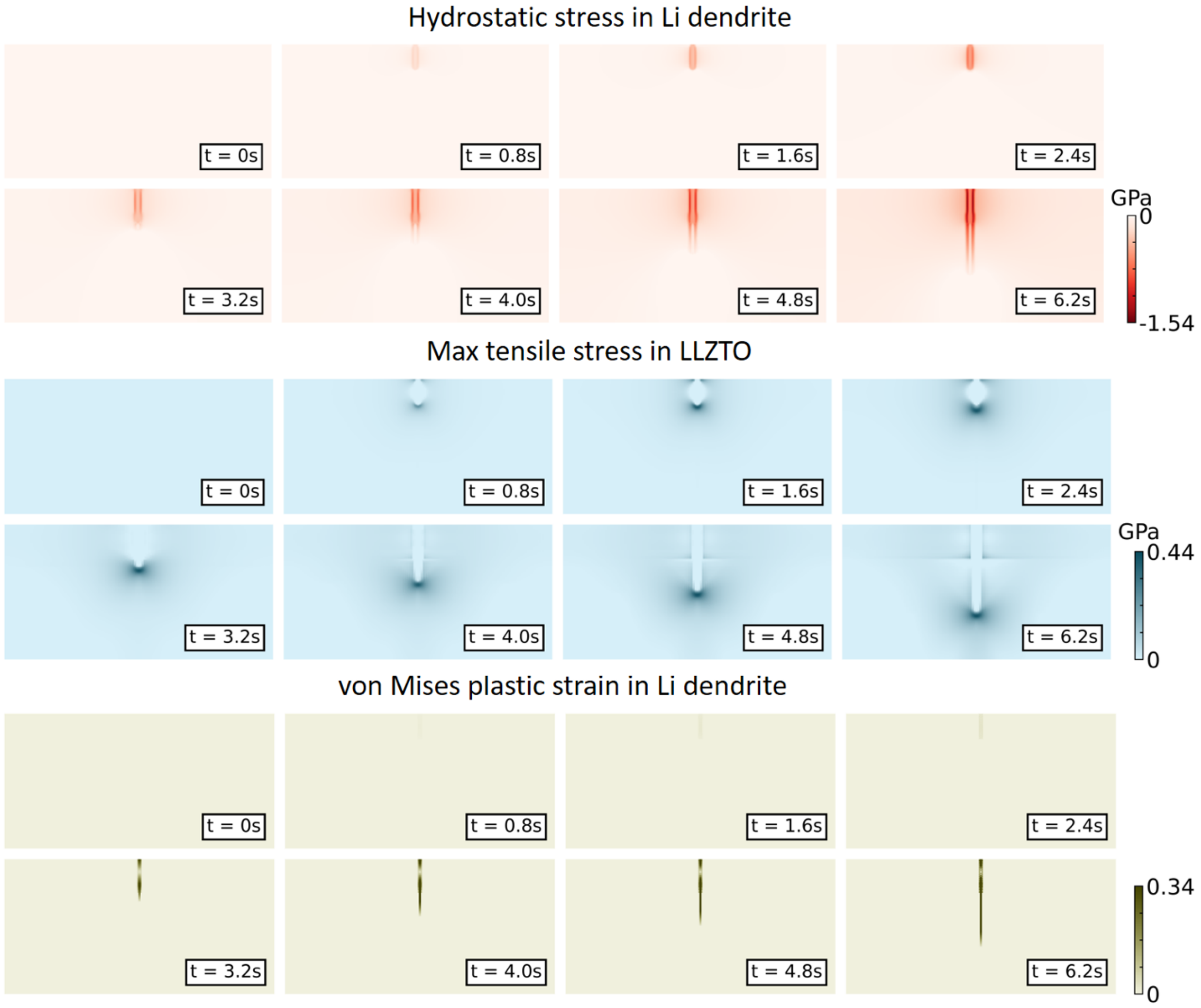

Figure S19. Distribution of hydrostatic stress in lithium dendrite, maximum tensile stress in LLZTO, and the von Mises plastic strain in lithium dendrite during lithium plating within LLZTO. The yield strength of lithium dendrite is assumed to be 1.25 MPa.

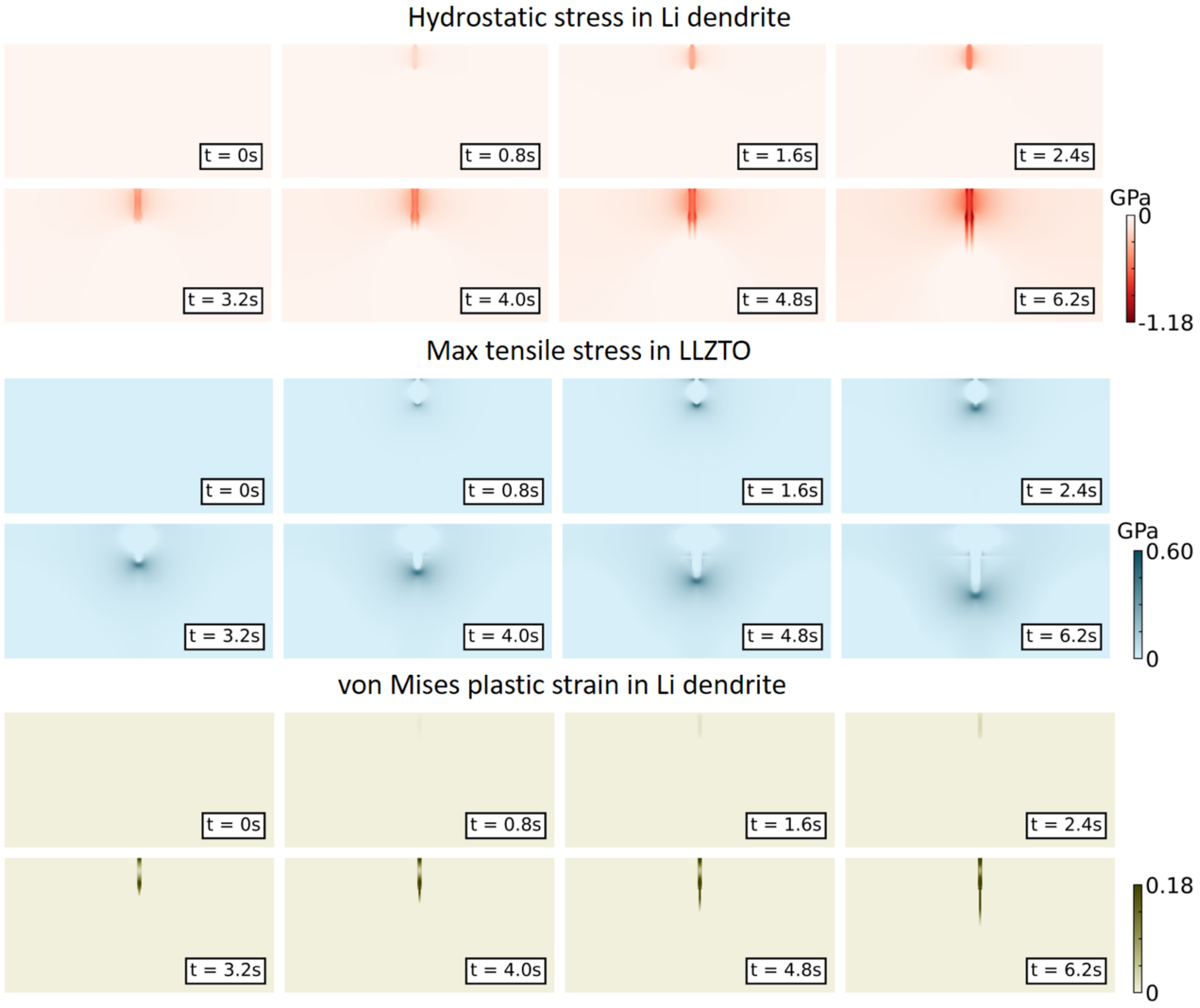

Figure S20. Distribution of hydrostatic stress in lithium dendrite, maximum tensile stress in LLZTO, and the von Mises plastic strain in lithium dendrite during lithium plating within LLZTO. The yield strength of lithium dendrite is assumed to be 12.5 MPa.

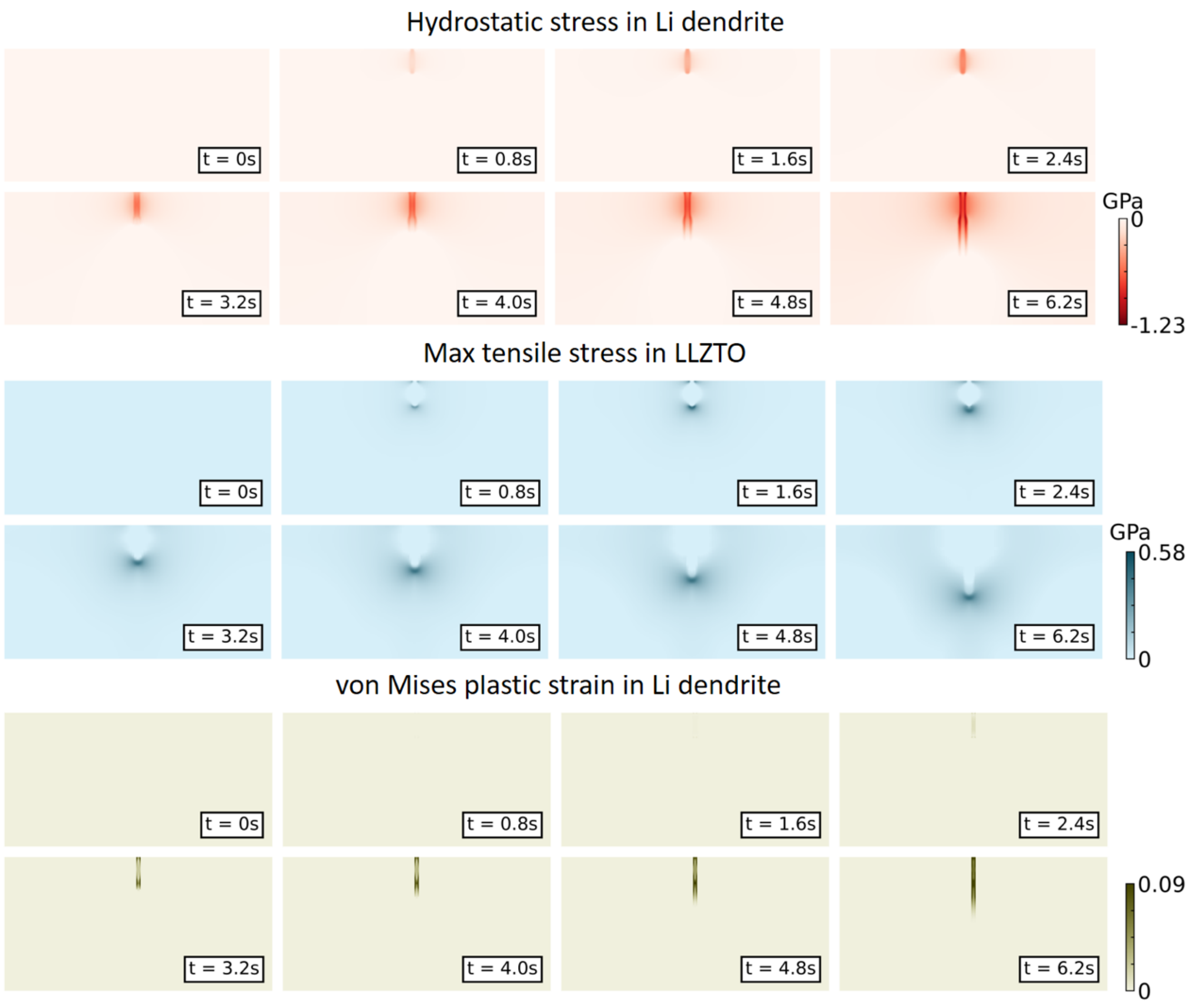

Figure S21. Distribution of hydrostatic stress in lithium dendrite, maximum tensile stress in LLZTO, and the von Mises plastic strain in lithium dendrite during lithium plating within LLZTO. The yield strength of lithium dendrite is assumed to be 62.5 MPa.

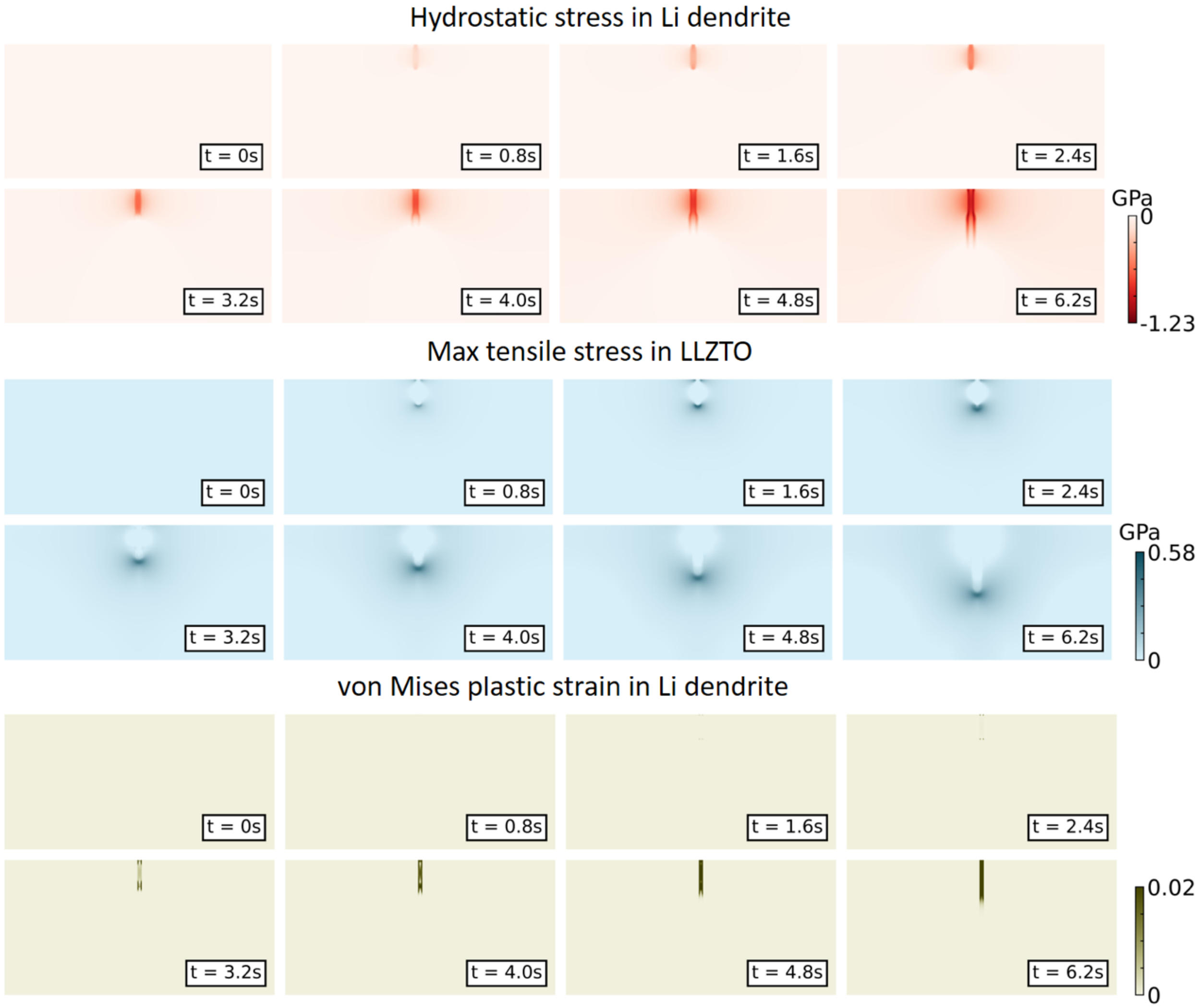

Figure S22. Distribution of hydrostatic stress in lithium dendrite, maximum tensile stress in LLZTO, and the von Mises plastic strain in lithium dendrite during lithium plating within LLZTO. The yield strength of lithium dendrite is assumed to be 125 MPa.

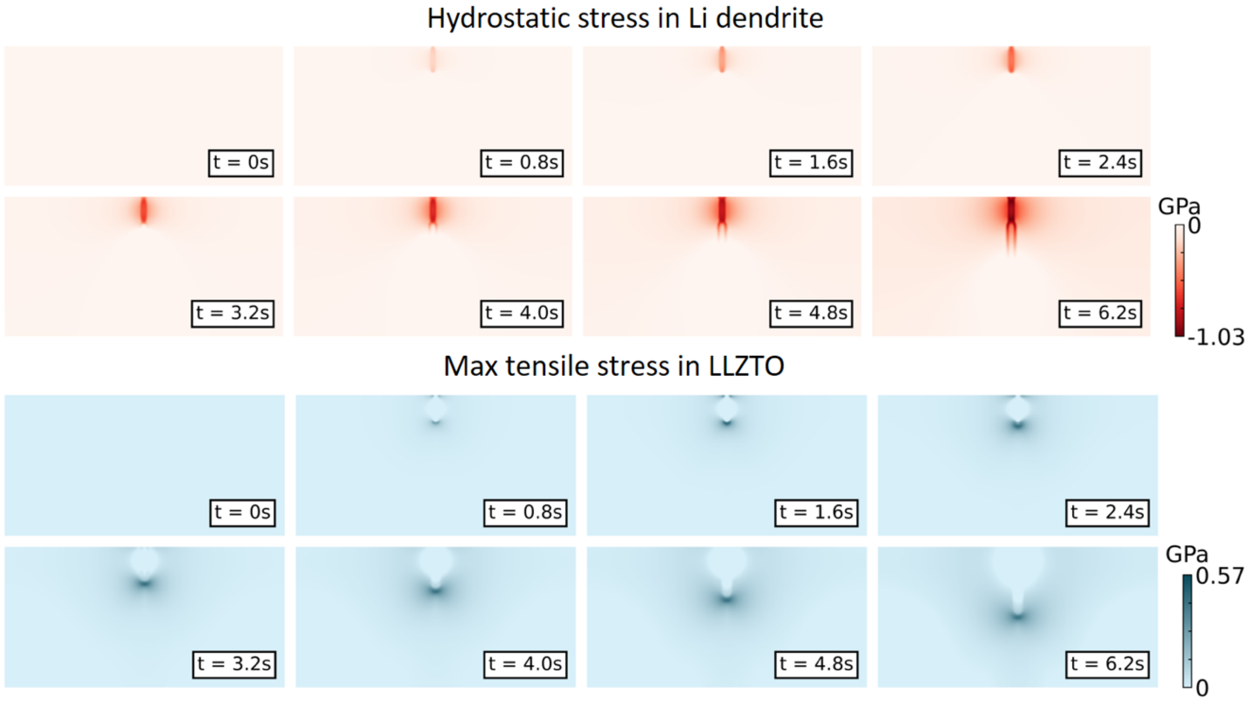

Figure S23. Distribution of hydrostatic stress in lithium dendrite, maximum tensile stress in LLZTO during lithium plating within LLZTO. The yield strength of lithium dendrite is assumed to be infinity (no plastic deformation).

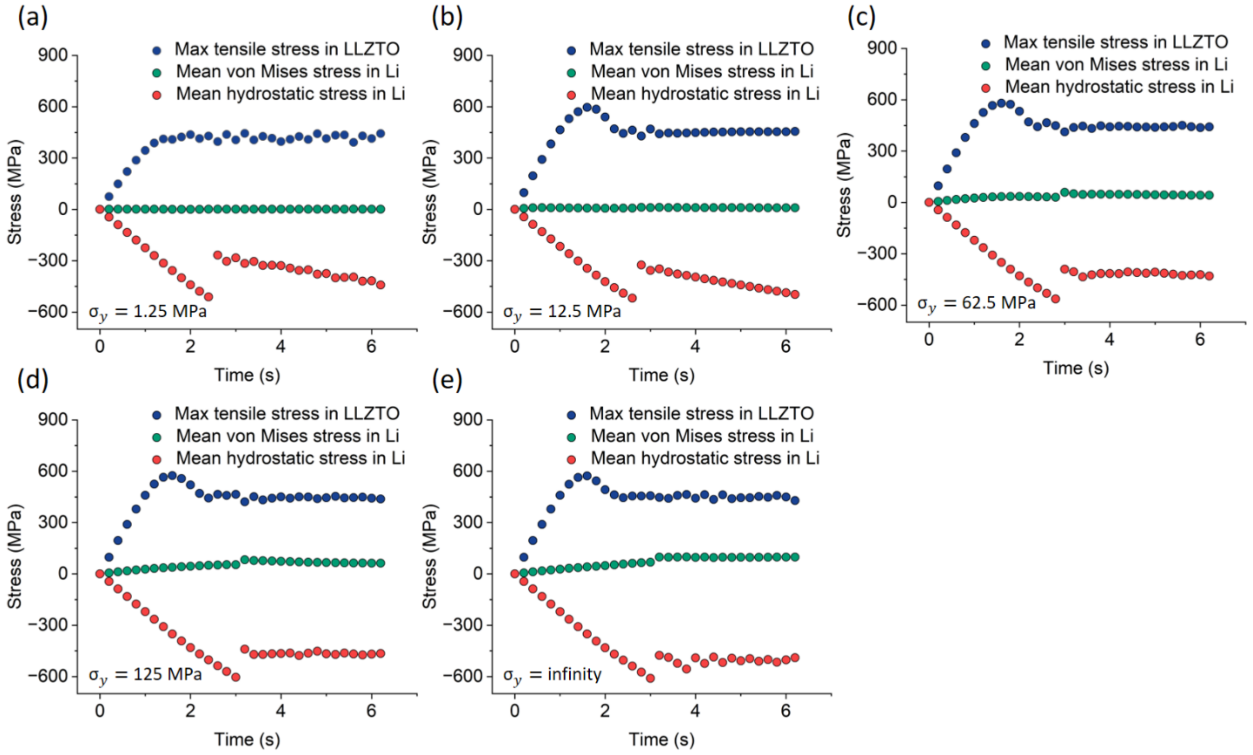

Figure S24. Time evolution of the maximum tensile stress in LLZTO ahead of the dendrite tip, the mean von Mises stress in lithium dendrite and the mean hydrostatic stress in lithium dendrite. The yield strength of lithium dendrite is assumed to be (a) 1.25 MPa, (b) 12.5 MPa, (c) 62.5 MPa, (d) 125 MPa and (e) infinity (no plastic deformation).

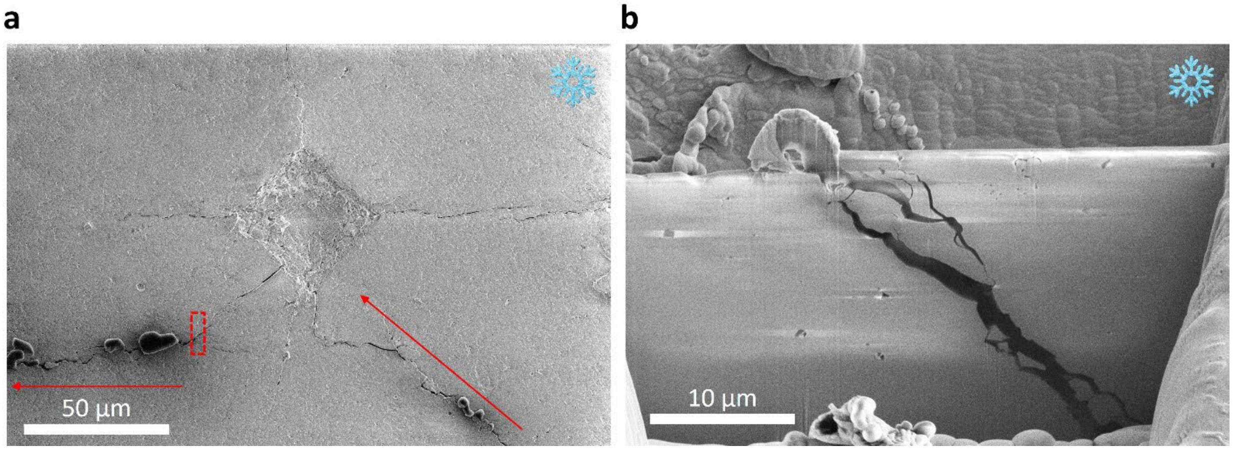

Figure S25. (a) Deflection of a lithium dendrite after interacting with Vickers indents. (b) Cross-sectional fractography of LLZTO induced by lithium plating in the region highlighted by the red square in (a). The region of interest was intentionally thinned down to ~ 100 nm to demonstrate that it is fully filled; otherwise, the background would be visible through the dry crack. Data source: in-plane cell.

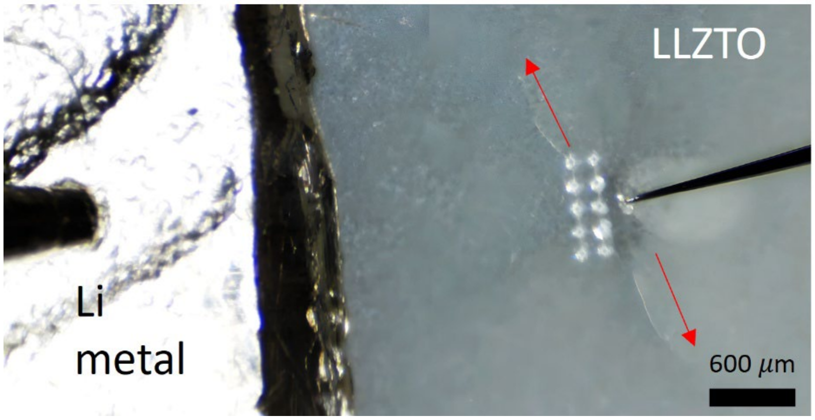

Figure S26. Additional evidence showing that the engineered voids can cause deflection of lithium dendrites. Data source: in-plane cell.

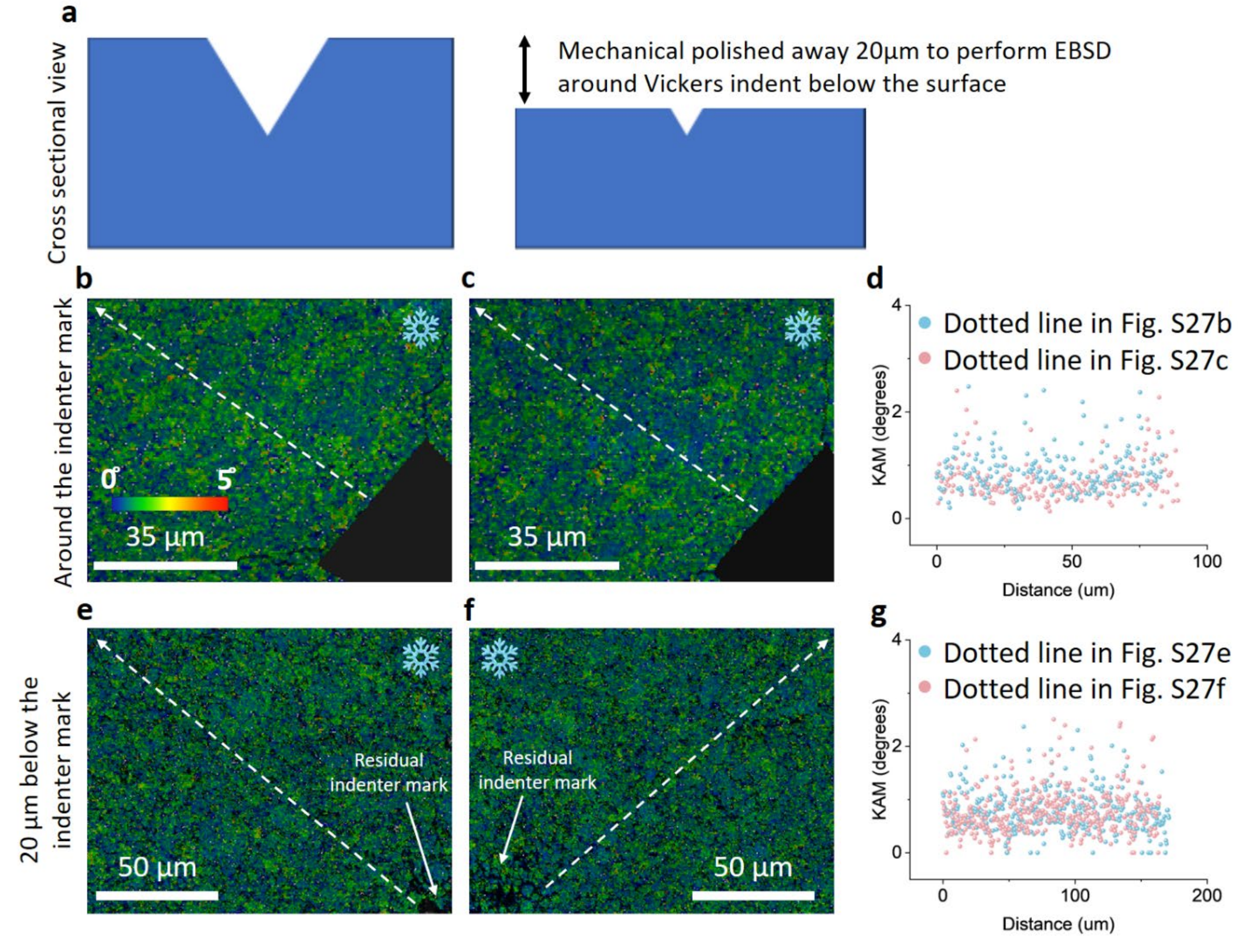

Figure S27. Kernel average misorientation (KAM) maps. (a) Schematic showing the sample preparation workflow for KAM mapping. (b-d) KAM maps of the regions around the Vickers indent. (e-g) KAM maps of the subsurface regions beneath the Vickers indent.

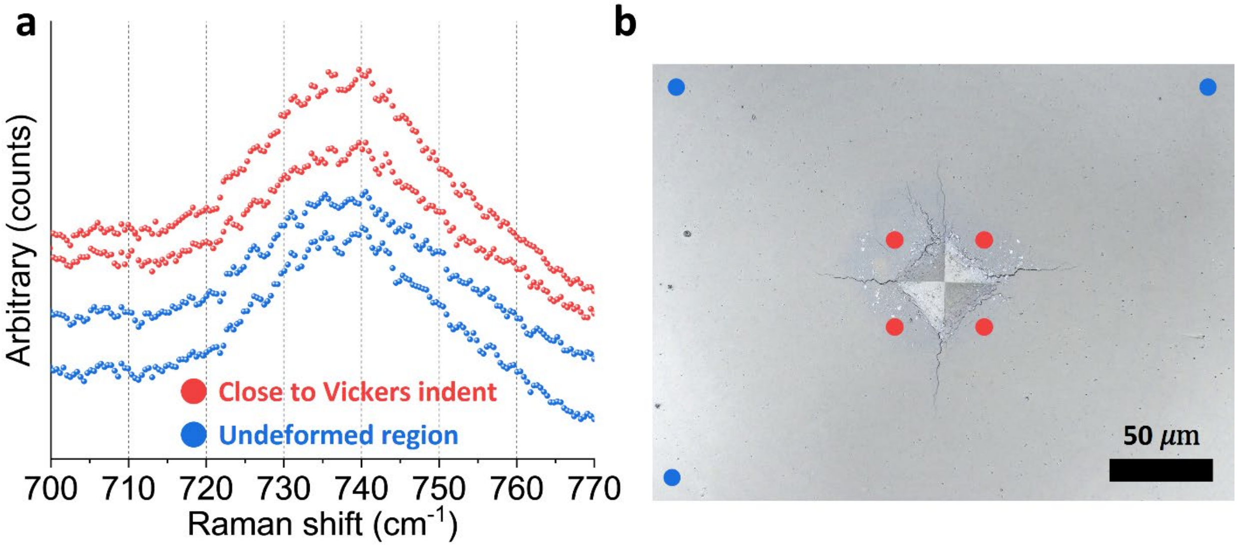

Figure S28. (a) Raman spectra collected from the region near the Vickers indent and from an undeformed reference area. Only two sets of data are shown, and the spectra are vertically shifted for clarity. (b) Optical micrograph showing the locations where the Raman measurements were performed. The red circles represent the scan near the Vickers indent, and the blue circles represent the scan in the undeformed region.

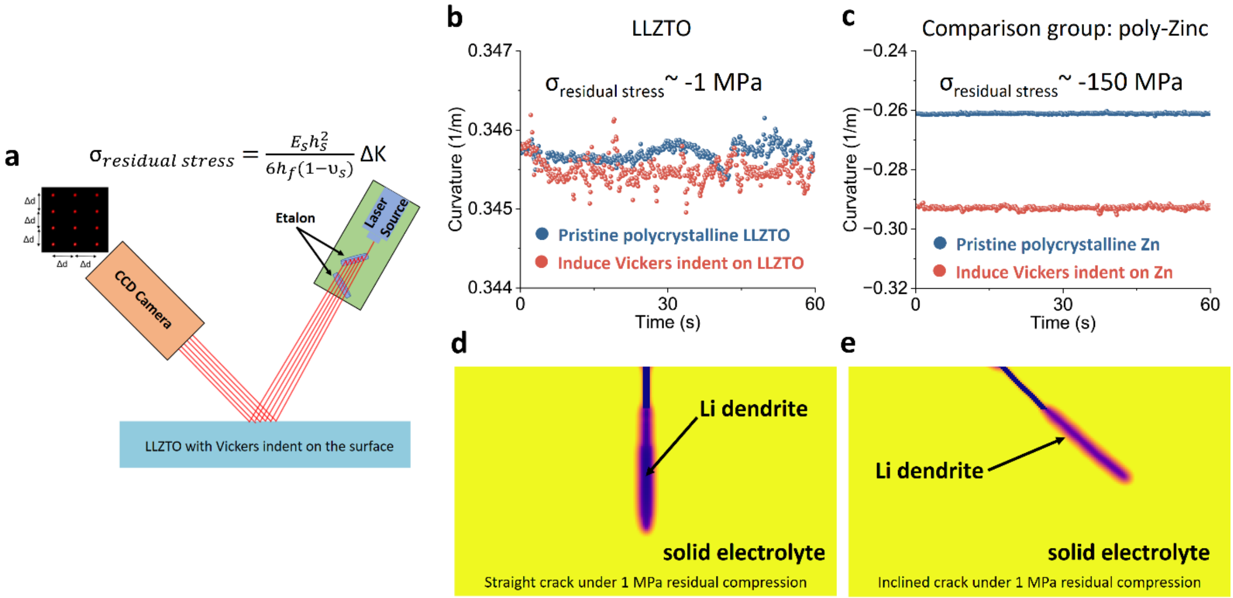

Figure S29. (a) Schematic of the multibeam optical stress sensor (MOSS) used to measure the residual stress induced by Vickers indentation. (b) Curvature changes of the polycrystalline LLZTO solid electrolyte before and after producing an array of Vickers indents (7 × 4 array with 150 µm spacing and 1 kg load). (c) Curvature changes of polycrystalline Zn before and after producing an array of Vickers indents (5 × 4 array with 200 µm spacing and 500 g load). This comparison highlights the markedly different residual stress levels typically found in brittle ceramics and ductile metals and validates the reliability of the MOSS method for capturing stress-induced curvature changes in both material classes. (d, e) Simulated lithium dendrite propagation paths under a residual compressive stress of 1 MPa, initiated from a vertical and an inclined notch, respectively.

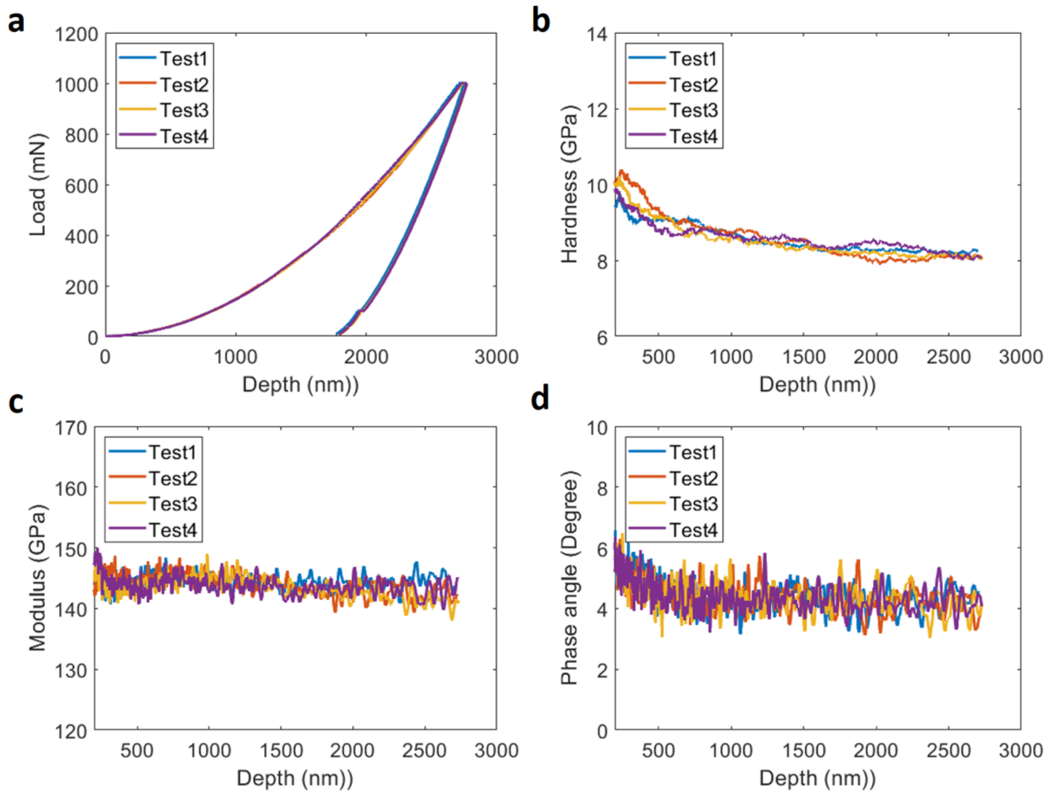

Figure S30 Nanoindentation results of polycrystalline LLZTO: (a) load-depth curve; (b) hardness-depth curve; (c) Young's modulus-depth curve; and (d) phase angle-depth curve.

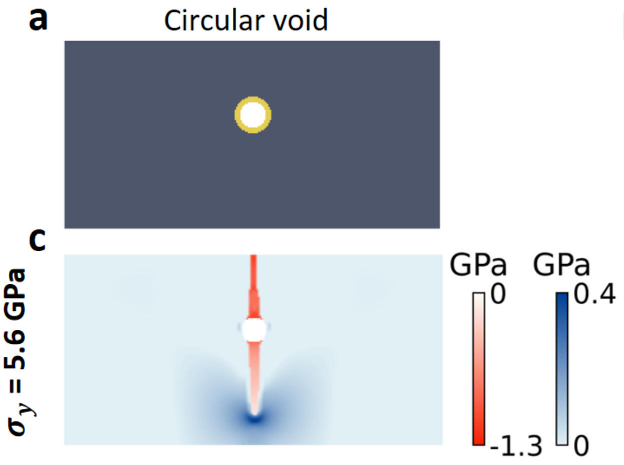

Figure S31. (a, b) Schematic illustration of the introduction of a thin annular shell surrounding the void. The shell thickness is 4 μm. The yield stress of the shell layer is 5.6 GPa. (c, d) Phase-field simulation of lithium dendrite interaction with (c) circular and (d) transverse voids embedded in the solid electrolyte, showing hydrostatic stress in lithium and maximum tensile stress in LLZTO during dendrite propagation.

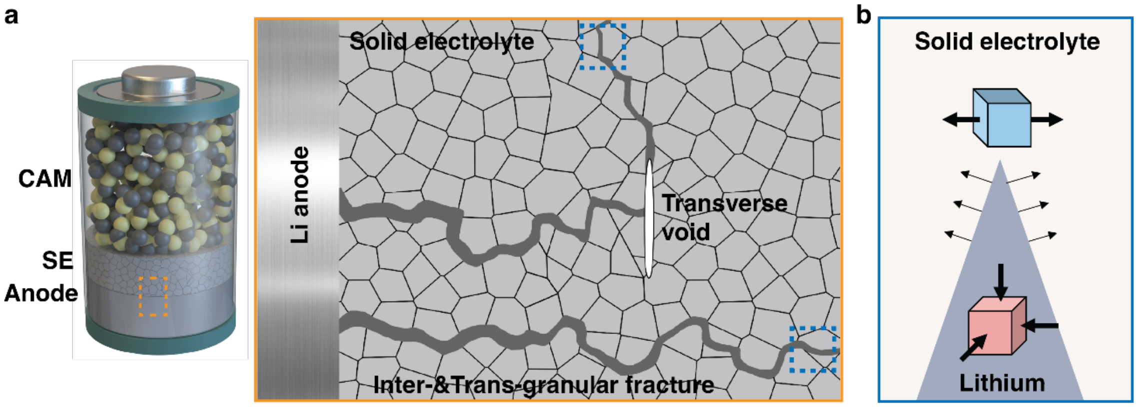

Figure S32. (a) Schematic of mechanically driven fracture during lithium dendrite propagation, leading to failure in solid-state batteries. (b) Schematic of the stress field in the lithium dendrite and solid electrolyte, with hydrostatic pressure buildup in lithium dendrite and tensile stress in the surrounding solid electrolyte. CAM: cathode active material; SE: solid electrolyte.

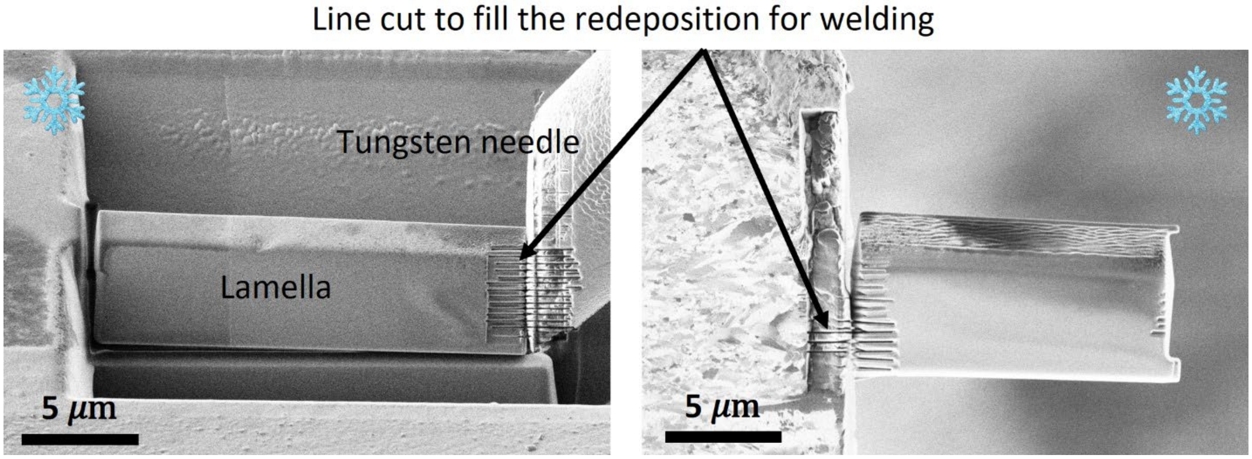

Figure S33. FIB lift-out technique performed at cryogenic temperature, utilizing redeposition effects induced by line cutting for attachment.

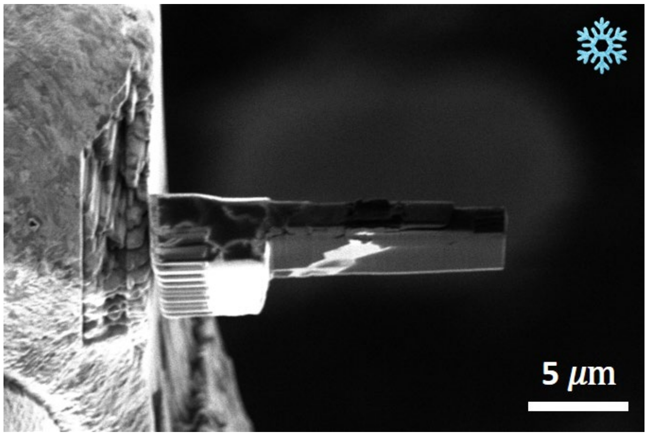

Figure S34. Ga^+ FIB lift-out lamella prepared for transmission Kikuchi diffraction (TKD) analysis. The lamella was maintained at a thickness of approximately 1 μm to ensure sufficient electron scattering and to preserve its mechanical integrity, thereby preventing bending or distortion-induced strain during ion-milling preparation. Data source: in-plane cell.

Movie S1. Full-length video (16× speed) showing lithium dendrite deflection induced by Vickers indents.

Reference

(1) Narayan, S.; Anand, L. On Modeling the Detrimental Effects of Inhomogeneous Plating-and-Stripping at a Lithium-Metal/Solid-Electrolyte Interface in a Solid-State-Battery. *Journal of The Electrochemical Society* **2020**, *167* (4), 040525. DOI: 10.1149/1945-7111/ab75c1.

(2) Yu, S.; Schmidt, R. D.; Garcia-Mendez, R.; Herbert, E.; Dudney, N. J.; Wolfenstine, J. B.; Sakamoto, J.; Siegel, D. J. Elastic Properties of the Solid Electrolyte $Li_7La_3Zr_2O_{12}$ (LLZO). *Chemistry of Materials* **2016**, *28* (1), 197-206. DOI: 10.1021/acs.chemmater.5b03854.

(3) Day, J. P.; Ruoff, A. L. The variation of the elastic constants of lithium with temperature and pressure. *physica status solidi (a)* **1974**, *25* (1), 205-213. DOI: https://doi.org/10.1002/pssa.2210250118.

(4) Fincher, C., Zhang, Y., Pharr, M. Measurements, Mechanisms, and Maps: The Mechanical Behavior of Lithium, Sodium, and Potassium Metal as a Function of Temperature, Strain-Rate, and Size (Under Preparation).

(5) Nonemacher, J. F.; Arinicheva, Y.; Yan, G.; Finsterbusch, M.; Krüger, M.; Malzbender, J. Fracture toughness of single grains and polycrystalline $Li_7La_3Zr_2O_{12}$ electrolyte material based on a pillar splitting method. *Journal of the European Ceramic Society* **2020**, *40* (8), 3057-3064. DOI: https://doi.org/10.1016/j.jeurceramsoc.2020.03.028.

(6) Wolfenstine, J.; Jo, H.; Cho, Y.-H.; David, I. N.; Askeland, P.; Case, E. D.; Kim, H.; Choe, H.; Sakamoto, J. A preliminary investigation of fracture toughness of $Li_7La_3Zr_2O_{12}$ and its comparison to other solid Li-ion conductors. *Materials Letters* **2013**, *96*, 117-120. DOI: https://doi.org/10.1016/j.matlet.2013.01.021.

(7) Lasia, A. The Origin of the Constant Phase Element. *The Journal of Physical Chemistry Letters* **2022**, *13* (2), 580-589. DOI: 10.1021/acs.jpclett.1c03782.

(8) Córdoba-Torres, P.; Mesquita, T. J.; Nogueira, R. P. Relationship between the Origin of Constant-Phase Element Behavior in Electrochemical Impedance Spectroscopy and Electrode Surface Structure. *The Journal of Physical Chemistry C* **2015**, *119* (8), 4136-4147. DOI: 10.1021/jp512063f.

(9) Lee, K.; Kazyak, E.; Wang, M. J.; Dasgupta, N. P.; Sakamoto, J. Analyzing void formation and rewetting of thin in situ-formed Li anodes on LLZO. *Joule* **2022**, *6* (11), 2547-2565. DOI: https://doi.org/10.1016/j.joule.2022.09.009.

(10) Krauskopf, T.; Hartmann, H.; Zeier, W. G.; Janek, J. Toward a Fundamental Understanding of the Lithium Metal Anode in Solid-State Batteries—An Electrochemo-Mechanical Study on the Garnet-Type Solid Electrolyte Li6.25Al0.25La3Zr2O12. *ACS Applied Materials & Interfaces* **2019**, *11* (15), 14463-14477. DOI: 10.1021/acsami.9b02537.

(11) Park, R. J. Y.; Eschler, C. M.; Fincher, C. D.; Badel, A. F.; Guan, P.; Pharr, M.; Sheldon, B. W.; Carter, W. C.; Viswanathan, V.; Chiang, Y.-M. Semi-solid alkali metal electrodes enabling high critical current densities in solid electrolyte batteries. *Nature Energy* **2021**, *6* (3), 314-322. DOI: 10.1038/s41560-021-00786-w.

(12) Sharafi, A.; Yu, S.; Naguib, M.; Lee, M.; Ma, C.; Meyer, H. M.; Nanda, J.; Chi, M.; Siegel, D. J.; Sakamoto, J. Impact of air exposure and surface chemistry on Li–Li7La3Zr2O12 interfacial resistance. *Journal of Materials Chemistry A* **2017**, *5* (26), 13475-13487, 10.1039/C7TA03162A. DOI: 10.1039/C7TA03162A.

(13) Athanasiou, C. E.; Fincher, C. D.; Gilgenbach, C.; Gao, H.; Carter, W. C.; Chiang, Y.-M.; Sheldon, B. W. Operando measurements of dendrite-induced stresses in ceramic electrolytes using photoelasticity. *Matter* **2024**, *7* (1), 95-106. DOI: 10.1016/j.matt.2023.10.014 (acccessed 2025/03/24).
